# Automated determination of fatty acids in soaps and detergents using the Technicon Auto Analyzer system

**DOI:** 10.1155/S1463924681000114

**Published:** 1981

**Authors:** A. Bedendo, P. Monaco

**Affiliations:** 1 Control Laboratory Mira Lanza Mira (Venice) Italy; 2 Technieon Italy Rome Italy


### Automated determination of fatty acids in soaps and detergents using the Technicon AutoAnalyzer system

A. Bedendo
*Control Laboratory, Mira Lanza, Mira (Venice), Italy*

P. Monaco

*Technicon Italy, Rome*

### Introduction

The quantitative determination of fatty acids in soap or other detergents is an important quality control parameter where products are cycled. Hence a fast, reliable and simple method of analysis is necessary, preferably automated. Two methods are routinely used for this assay:

1. A solvent extraction method using petroleum ether.
2. A water evaporation technique.

Both methods are tedious, very prone to error and involve constants dependent on the manufacturing process.

A more rapid and simple method has been described [1,2] in which the saponified fatty acids in the aqueous solution are titrated with an organic, water immiscible solution of a quaternary ammonium salt (QA) complexed with bromocresol green (BCG) used as an indicator. The end point of the titration is taken as that point where the colour intensity of the two liquid phases is the same.

Given certain pre-requisites, the authors considered that this technique could be automated using a continuous flow system.

### Experimental

In order to develop the automated method the reaction was first studied using the manual method.

### Principle

The reaction used consists of an exchange between the soap in the sample and a bromcresol green complex with quaternary ammonium salt. This reaction frees an equivalent of BCG for each equivalent of soap involved in the reaction. While the BCG complex is soluble in a chloroform isopropanol mixed solvent, the free dye is insoluble and consequently migrates into the water layer. As a result, the sample causes decolouration of the organic layer which can be measured colorimetrically. The results quoted by Milwidsky and Holtzmann [2] certainly suggest that the equilibrium point of the reaction is strongly to the right, ie towards the reaction products – QA/fatty acid complex plus free BCG. A colorimetric measurement of either the organic or aqueous phase would, therefore, produce a Beer's Law relationship between the concentration of the fatty acid and the optical density of measured colution.

### Preliminary investigations

Using an equimolecular solution of trimethylcetyl ammonium bromide and BCG in a 4:1 mixture of chloroform/isopropanol, the optical density of both the organic and aqueous phase was determined when an equal volume of water containing varying concentrations of fatty acid were mixed. The optical density was linear with the concentration of fatty acid.

During these investigations, the following points were noted:

1. To obtain reproducible results, the organic solution must be prepared with care and to an exact optical density.
2. Properly prepared, the organic phase is stable at room temperature for at least 10 days.
3. Over 10 days the calibration curves do not change suggesting no appreciable change in the equilibrium constants.
4. The alkalinity of the aqueous solution is critical. With sodium hydroxide concentration below 3%, the organic phase becomes very turbid, decreasing in turbidity until at 10% it is quite clear. Higher percentages lead to difficulties with the continuous flow system. Alkaline buffers, other than sodium hydroxide proved unsatisfactory.
5. The presence of ethyl alcohol in excess of 5% in the aqueous phase apparently displaced the reaction equilibrium – probably by altering the partition co-efficient between the aqueous and organic phase and leading obviously to erroneous results.
6. Erroneous results were also obtained when aqueous solutions contained an excess of strong electrolyte such as potassium chloride at a concentration of 5 g/l.

### Automation

Technicon AutoAnalyzer I and AutoAnalyzer II equipment was used comprising Sample II or IV, Pump I or III, and, in each case, a colorimeter with 15 mm flowcell and 660 nm filters and a recorder. All glassware, connections and pump tubes were standard Technicon products.

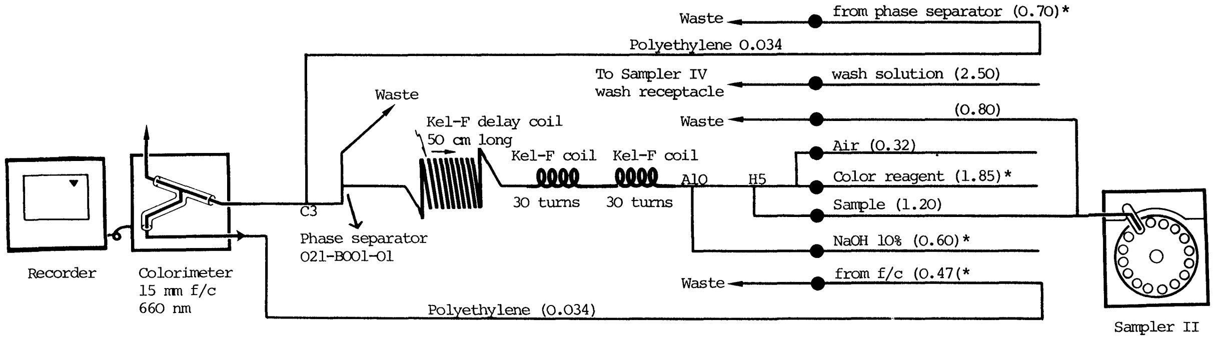

Note: Figures in parentheses represent flow rates in ml/min

* Acidflex tubing

*Figure 1. Flow diagram for the AutoAnalyzer I fatty acid in soap method (Range: 30–45 ppm)*

Figure 1 shows the flow diagram for the AutoAnalyzer I system and Figure 2 the flow diagram for the AutoAnalyzer II system. Features of the hydraulic system which should be highlighted are:

1. In place of the glass bead filled coils normally recommended by Technicon for solvent extraction, Kel-F tubing, 1.5 mm bore by 1.5 m in length wound into a coil of 3 cm in diameter was substituted.
2. Into the phase separator (Technicon No. 021-B001-01) a teflon strip was inserted to increase the separation efficiency of the organic and aqueous phase. However, in place of the nipple connector to the tubing Acidflex tubing was slipped over the arms of the separator. The nipple connector obstructed – the flow at a point where smooth flow was essential (Figure 3).

Further important modifications to normal AutoAnalyzer operation are:

1. There must be no wetting agent in the organic phase which is measured. The aqueous soap phase provides sufficient lubrication.
2. Since no air segmentation is used, the inter-sample bubble must be removed (see Figures 1 and 2).

**Table 1**

| Sample No | Technicon AA-I, first generation 20 samples/ hour (40 ppm) | Technicon AA-I, first generation 30 samples/ hour (40 ppm) | Technicon AA-II, second generation 20 samples/ hour (30 ppm) | Technicon AA-II, second generation 50 samples/ hour (30 ppm) |
|---|---|---|---|---|
| 1 | 39.60 | 40.00 | 30.27 | 30.09 |
| 2 | 39.60 | 40.17 | 30.27 | 29.41 |
| 3 | 38.88 | 40.56 | 30.62 | 30.09 |
| 4 | 38.88 | 39.39 | 31.51 | 29.41 |
| 5 | 39.96 | 39.78 | 30.97 | 30.6 |
| 6 | 38.88 | 40.56 | 31.33 | 29.75 |
| 7 | 39.24 | 39.78 | 31.68 | 30.09 |
| 8 | 38.88 | 40.00 | 31.15 | 30.43 |
| 9 | 40.32 | 41.34 | | 30.09 |
| 10 | 38.88 | 41.34 | | 30.60 |
| Mean | 39.30 | 40.30 | 30.97 | 30.05 |
| SD | 0.53 | 0.60 | 0.54 | 0.40 |
| CV% | 1.34 | 1.52 | 1.75 | 1.40 |

### Reagents

1. Cetavlon (trimethylcetyl ammonium bromide, 0.0034 M) was obtained from Carlo Erba, Milan, Italy. 1.25 g Cetavlon is dissolved in 200 ml isopropanol in a one litre volumetric flask. The solution is made up to one litre with distilled water.
2. BCG was obtained from E Merck, Darmstadt, Germany. 0.05 g BCG is dissolved in approximately 100 ml of distilled water. One pellet of solid sodium hydroxide and 10 ml of isopropanol are added to a one litre volumetric flask. The solution is made up to one litre with distilled water.
3. The organic solvents were obtained from E Merck, Darmstadt, Germany. 200 ml of isopropanol are added to 800 mil of $CHCl_3$.
4. Coloured complex solution. A one litre separating funnel is filled with 600 ml of organic solvent (a mixture 4:1 of chloroform isopropanol, 100 ml BCG solution (2) and 15 ml Cetavlon solution (1). The separating funnel is shaken and inverted at least 50 times. The organic phase is drained off, filtered on a Whatman 40 filter paper and stored in an amber bottle. Before use, the solution must be checked for optical density. The spectrophotometer must be zeroed on solution (3). Solution (4) must be 0.43 ± 0.04 OD units with a 1 cm cell.
5. 10% w/v aqueous solution sodium hydroxide.
6. Solution of sodium hydroxide, pH 12. To one litre of distilled water, 15 ml of sodium hydroxide solution are added.

### Calibration

Fatty acid of known composition and known molecular weight was saponified by alcoholic sodium hydroixde using standard methods. Standards of free fatty acid were prepared in the range 0-50 ppm by diluting the appropriate volume of saponified soap in pH 12 sodium hydroxide solution.

### Sample

One gram of soap sample, accurately weighed, is dissolved in a one litre volumetric flask using 50% ethyl alcohol with heating (if necesssary) and stirring. It is made up to one litre with 50% ethyl alcohol solution. The solution is then diluted in pH 12 sodium hydroxide according to the anticipated range of free fatty acid (60% - 25 ml in 500 ml; 80% - 20 ml in 500 ml). The dilution factors are only approximate; they are based on an average molecular weight of 250.

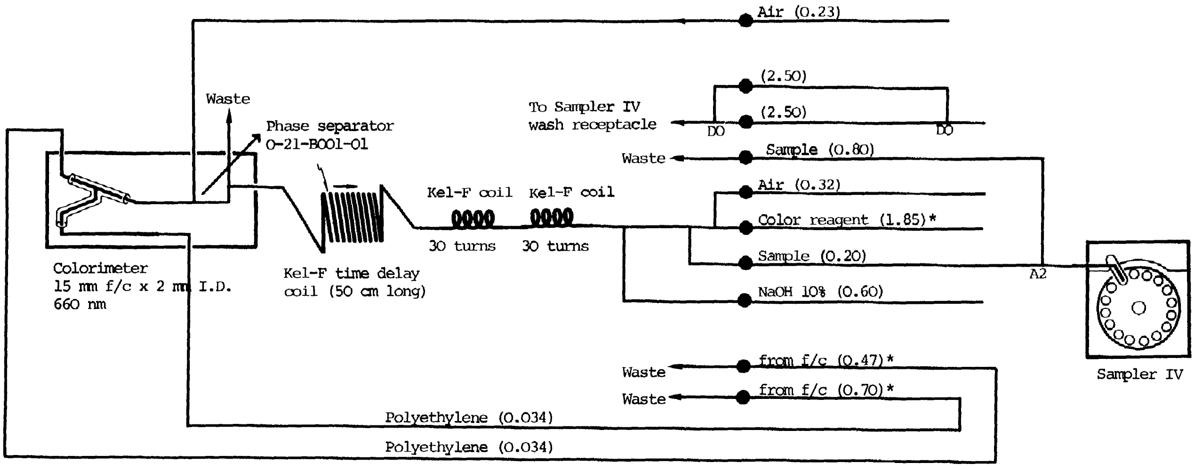

Note: Figures in parentheses represent flow rates in ml/min

* Acidflex tubing

*Figure 2. Flow diagram for the AutoAnalyzer II fatty acid in soap method (Range: 25–35 ppm)*

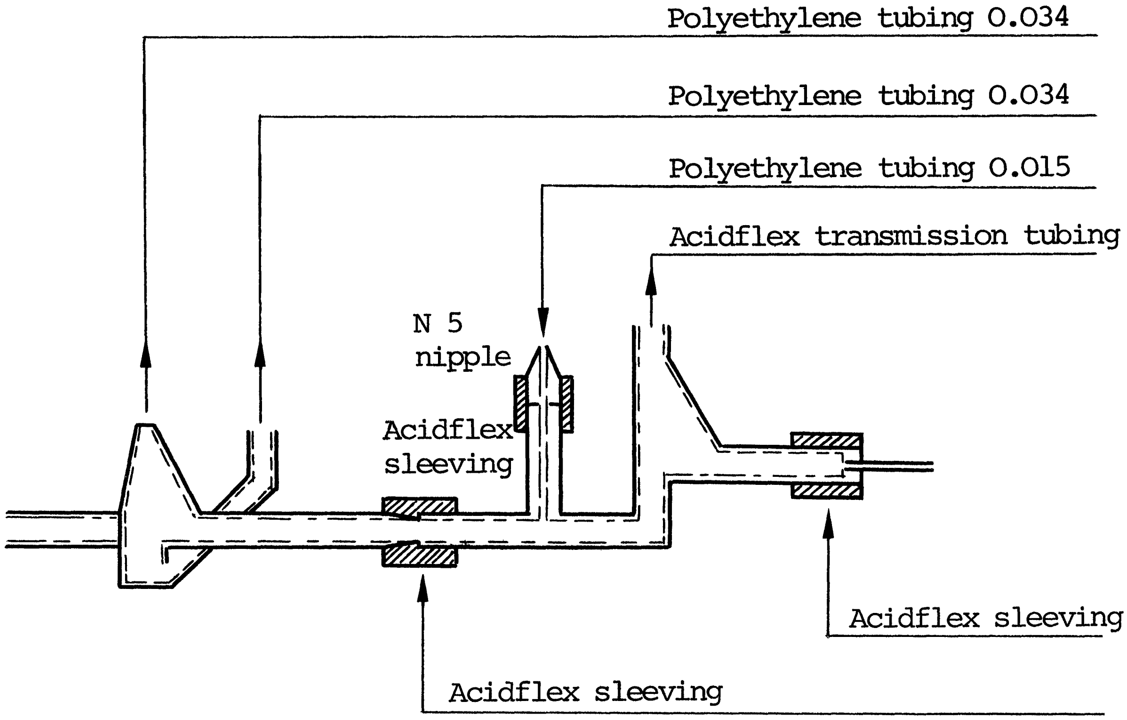

*Figure 3. Phase separator assembly*

### Calculation of results

Peak heights were compared to those of the standard solution and the free fatty acid concentration was calculated on the basis of linearity between two standards.

### Discussion

As many AutoAnalyzer I units are still in operation, the method developed was tested on both AutoAnalyzer I and AutoAnalyzer II systems.

The standard deviation and the coefficient of variation have been calculated from the heights of the standard 30 ppm and 40 ppm peaks obtained at analytical frequencies of 20/30/50 samples/hour. The standard in question had an average fatty acid molecular weight of 250. Results are shown in Table 1. In order to obtain correct results, it is necessary to know the average molecular weight of fatty acids in the sample. That is, the samples analysed must be compared with standards in the calibration curve with more or less the same average molecular weight.

It should be noted that the method is reliable for samples with an average molecular weight of 200 to 300, but not outside this range. Preferably, the molecular weights of fatty acids within the same sample should not vary by too much.

Interestingly, increasing the sample rate on the generation I AutoAnalyzer from 20-30 samples/hour decreases precision while increasing the rate on generation II from 20-50 samples/hour increases the precision. The faster change of sample in the AutoAnalyzer II prevents the build up of deposits in the significantly shorter hydraulic path as compared to the very long hydraulic paths used in the older AutoAnalyzer I systems.

It must be emphasised, however, that a single calibration is valid for free fatty acids within a limited molecular weight. However, experience has been limited to molecular weights between 200 and 300 – a normal range in soap manufacture.

REFERENCES

[1] Epton S. R., *Nature*, **160**, 795, 1947: Frans Faraday Soc, **44**, 226, 1948

[2] Milwidsky, B. M. and Holtzmann S., *Soap and Chemical Specialities*, **XLII**, 5, 83, 1966

